# Harmonizing HeLa cell cytoskeleton behavior by multi-Ti oxide phased nanostructure synthesized through ultrashort pulsed laser

**DOI:** 10.1038/srep15294

**Published:** 2015-10-15

**Authors:** Chandramouli Chinnakkannu Vijayakumar, Krishnan Venkatakrishnan, Bo Tan

**Affiliations:** 1 Ultrashort laser nano manufacturing research facility, Department of Mechanical and Industrial Engineering, Ryerson University, 350 Victoria Street, Toronto, ON, Canada, M5B 2K3; 2 Nano imaging lab, Department of Aerospace Engineering, Ryerson University, 350 Victoria Street, Toronto, ON, Canada, M5B 2K3

## Abstract

Knowledge about cancer cell behavior on heterogeneous nanostructures is relevant for developing a distinct biomaterial that can actuate cancer cells. In this manuscript, we have demonstrated a harmonized approach of forming multi Ti-oxide phases in a nanostructure (MTOP nanostructure) for its unique cancer cell controlling behavior.Conventionally, single phases of TiO_2_ are used for targeted therapy and as drug carrier systems.In this research, we have shown a biomaterial that can control HeLa cells diligently using a combination of TiO, Ti3O and TiO2 phases when compared to fibroblast (NIH3T3) cells.MTOP-nanostructures are generated by varying the ionization energy in the vapor plume of the ultrashort pulse laser; this interaction with the material allows accurate tuning and composition of phases within the nanostructure. In addition, the lattice spacing of MTOP-nanostructures was analyzed as shown by HR-TEM investigations. An FESEM investigation of MTOP-nanostructures revealed a greater reduction of HeLa cells relative to fibroblast cells. Altered cell adhesion was followed by modulation of HeLa cell architecture with a significant reduction of actin stress fibers.The intricate combination of MTOP-nanostructures renders a biomaterial that can precisely alter HeLa cell but not fibroblast cell behavior, filling a void in the research for a biomaterial to modulate cancer cell behavior.

Regulating cancer cell behavior is a complex biological process, in which there is a need to restrain the cytoskeletal arrangement by bio-mimetic nano structured materials^1^,^2^,^3^. This communication is mediated by the direct interaction between cell surface receptors and physical extra cellular matrix (ECM) molecules. The mnemonic ability of these ECM’s plays an imperative role in regulating cancer cell behavior such as cell adhesion, spreading, proliferation, differentiation, gene expression and signal transduction^4^,^5^,^6^. Two approaches that can synthesize the physical ECM are creating a biocompatible surface nanostructure topography and composing hydrophilic functional groups on nanostructures to initiate a specific cellular response.

The dialogue between surface nanostructure materials and cancer cell behavior is a consequence of an external biophysical stimulus, which is crucial for understanding many fundamental biological questions in designing biomaterials^7^. Anodization is one of the primary techniques that can synthesize the surface nanostructure topography^8^. Currently many experimental evidences indicate that the cell spreading has been achieved due to an increase in focal adhesion formation on nanotubes^9^. However, the aforesaid research work has no control over the construction of such nanotubes having uniform material composition. On account of an extended anodization process their outermost tubes not only substantially become thinner but also disintegrate^10^. As a result, the nanotube feature size and material composition are of great concern in regulating cancer cell behavior.

Functionalizing the titanium nanotubes by combining them with drug delivery systems, including functional proteins^11^, growth factor enclosed in fibrin hydrogels^12^ and anti-inflammatory drug-eluting compounds^13^ determines the cell fate, cell adhesion, proliferation and differentiation. Commonly used methods for surface coating the titanium nanotubes are sol-gel, evaporation-induced surface crystallization and spin assisted layer-by-layer technique^14^,^15^,^16^. For instance, Mehdi Kazemzadeh-Narbat *et al.,* reported that the phospholipid coating of CaP in titania nanotubes shows a reduction in bacterial growth but allows proliferation of bone cells. However, the limitations on loading capacity of the drug onto the nanotubes is dependent on the structural parameters such as the tube diameter and length^17^. In order for cells to interact with nanotubes, their optimum diameter is 15nm, which indicates the limitations in releasing a drug for a longer duration^18^,^19^.

Titanium is a common and well-established biomaterial that is widely used in dental, orthopedic and cardiovascular implants because of its biocompatibility, biodegradability, strong chemical stability and mechanical strength^20^. Also titanium is now being proposed to be used as a carrier for drugs and to reduce magnetic resonance imaging artifacts^21^.When titanium is exposed to the atmosphere, a protective TiO_2_ stable oxide film is formed that provides the ability to adsorb proteins due to the high surface to volume ratio, thereby inducing a platform for cells to differentiate. These findings imply that amongst the various approaches to modulate cell migration behavior, prominent are by varying the nanotopography of the substrate^2^,^18^,^22^, controlling spacing in between the nanostructures and drug loading of nanotubes^23^,^24^. These approaches have brought to light the numerous intrinsic investigations possible and in understanding the factors governing cell adhesion onto the nanostructure which are incidentally limited to specific morphology such as nanotubes, nanorods and nanowire. There are no reported studies where the nanostructures’ size has been retained without variation, while varying the material composition of the nanostructure to study different possible effects responsible for cancer cell behavior^2^. In particular, designing an effective nanostructure that governs HeLa cell cytoskeletons but allows mammalian cells to spread has remained a fundamental challenge^20^.

The objective of the current research is to generate the combinatorial titanium oxide phased nanostructure that selectively allows fibroblast cells to proliferate but controls the differentiation of HeLa cancer cells (Fig. 1). Ultrashort laser pulses allow the formation of various combinations of multi-Ti oxide phases in a nanostructure, which cannot be achieved through any other fabrication method. The ionization energy of the laser pulses and pulse-to-pulse separation time were able to induce various concentrations of multi-Ti oxide phases in the nanostructure. The results at a high ionization energy at the shortest pulse-to-pulse separation time displayed a material chemistry property of a rutile phase dominance along with the presence of a hongquiite phase in this multi-Ti oxide phased nanostructure. The dominant rutile phase with the presence of hongquiite phase nanostructures resulted in the non-proliferation of the HeLa cancer cells but simultaneously allowed proliferation of fibroblast cells. Thus, the consequence of the generation of multi-Ti oxide phase nanostructures has opened up the possibility to judiciously alter the entire material chemistry of these multi-Ti oxide phased nanostructure while at the same time retaining the continuous formation of the nanostructure.

## Materials and Methods

### Synthesizing multi-Ti oxide phased nanostructure formation through ultrashort pulsed laser

The simple but effective tuning of multi-Ti oxide phased nanostructures with pattern regularity is achieved through ultrashort pulsed laser interactions. Titanium samples (ASTM B265) were ground finished by grit silicate carbon paper to remove macro level defects and finally machine polished using alumina. The irradiation from the ultrashort pulsed laser source was constituted by a 1040 nm wavelength direct-diode-pumped Yb-doped fiber amplified femtosecond laser system (Clark MXR) with an average power of 16W and repetition rate ranging from 4 MHz to 26 MHz.The titanium sample was mounted on a precision X-Y-Z stage normal to the ultrashort pulsed laser beam.

The ultrashort pulsed laser interaction technique is a unique non-contact nanostructure processing approach that can facilitate in synthesizing various combinations of multi-Ti oxide phases in nanostructures through the vapor condensation mechanism, where the material undergoes a phase transformation through melting and vaporization as well as phase explosion if the laser fluence is sufficiently high.

### Cell culture

HeLa cells, a human cervical cancer cell line, were obtained from ATCC (American Type Culture Collection, ATCC No. CCL-2) and were cultured in DMEM-F12 medium with phenol red containing 10% heat inactivated fetal bovine serum and 1% penicillin-streptomycin at 35 °C in 5% CO_2_. NIH3T3, fibroblast cells was grown in DMEM medium containing 10% heat activated fetal bovine serum with 1% penicillin-streptomycin antibiotics at 35 °C in 5% CO_2_.

### Scanning electron microscopy and transmission electron microscopy

The specimen of the multi–Ti oxide phase nanostructure was qualitatively evaluated using a field emission–scanning electron microscope (Hitachi, SU-8200), and HeLa cells seeded onto the sample were analyzed using a scanning electron microscope (Hitachi, SU-1500). To study the interaction of cells with the nanostructure, the cells were incubated at 24 hours and 48 hours. After incubation, the spent medium was removed and the sample was fixed by glutaraldehyde for 30 minutes at 4 °C. Subsequently, the sample was washed twice with 1% sodium cacodylate buffer at 4 °C. Then, the cells were dehydrated through a graded ethanol series (from 10% to 100%) for 15 min. Then, the samples were critical point dried and prior to SEM examination, samples were sputtered with a gold layer. The individual nanoparticle analysis was performed using transmission electron microscopy (Hitachi H7000 HR-TEM). Energy dispersive X-ray spectroscopy (EDS) was performed to study material composition of the multi-Ti oxide phased nanostructure.

### X-ray diffraction

The elemental composition data of multi-Ti oxide phase nanostructure was collected on Bruker AXS D8 Advance micro diffraction system equipped with Cu-Ka source and a graphite monochromatic for the elimination of unwanted Cu-K-beta lines. The interaction of the incident rays with the sample creates an interference pattern and the diffracted X–rays are detected, processed and scanned through a 2D detector. All the possible lattice planes were obtained by calculating the d-spacing, thus allowing us to identify the individual mineral. Each mineral has a signature d-spacing, which is compared with the standard patterns for identifying the phase corresponding to the diffraction peaks.

### Fluorescent staining of cells

The samples are first fixed in paraformaldehyde, followed by incubation in milk to prevent non-specific binding. To stain the actin and cytoskeleton, the samples are incubated with Alexa Fluor 488(Life Technologies) followed by DAPI (4′, 6′-diamidino-2-phenylindole, Life Technologies) to stain the nucleus. An epi-fluorescent Nikon E-400 microscope was used and the data were recorded by a DS-5M-U1 color digital camera (Nikon, Canada).

### AFM analysis

An AFM was used to measure the phase contrast and topographical contour mapping of the titanium nanomaterial and their interaction with HeLa cell. They were mapped in the non–contact mode using NT-MDT, AFM, Russia equipped with prefabricated cantilever at scan rate of 30 min and all images were acquired at a scale of 100 × 100 μm for better resolution. All of the images obtained by AFM were measured using the same cantilever and identical scanning conditions.

### Statistics

All experiments were done in triplicate, and the data represent the mean ± standard deviation unless otherwise mentioned.

## Results and Discussions

### Synthesizing multi-Ti oxide phased nanostructures using ultrashort pulsed laser

A laser ablation plume is a highly dynamic and non-linear process. The interaction of plume in the presence of ambient gas is a highly elaborate gas dynamic process involving various steps: surface adsorption of laser, vaporization, plasma ignition, plasma adsorption, and rapid cooling followed by condensation^25^. When the energy delivered by the ultrashort pulsed laser is in excess of the binding energy of that atom, it breaks by means of repetitive laser pulses. When the ultrashort pulsed laser interacts with the target material, the surface is heated to a higher temperature and they combine with air that is present in the background to obtain the energy required for vaporizing the material: thus, the plasma plume is formed, consisting of electrons and ionized atoms. When this plasma plume expands outward, there is a heat transfer between the plume and the ambient gas, resulting in a cooling of plume and the condensation process begins. This condensation process results in a nucleation step, where the growth of the supercritical nuclei is initiated and comes to a halt by quenching. Thus, once the growth of nanoparticles is initiated, they aggregate due to the collision of nuclei and hence 3-D nanostructures are formed. When these ultrashort laser pulses are focused onto the target material, only the localized region within the focal volume absorbs the energy by a nonlinear process, such as multiphoton avalanche ionization, resulting in minimizing the thermal stress and collateral damage. The plume diffusion time depends on the various conditions: laser parameters, material properties and the ambient gas condition^26^. Earlier studies revealed that only MHz laser pulse repetition rates can keep the atomic flow into the plume at the critical level to synthesize 3-D nanostructures^27^,^28^.

The evaporation rate of a single pulsed laser can be expressed as


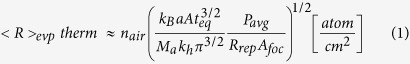


The number of atoms evaporated from a single spot after multiple laser pulses interact in a time interval D_t_ is expressed as


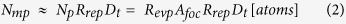


Therefore, by substituting Eq. 1 into Eq. 2, the number of atoms evaporated by multiple laser pulses based on laser parameters and the material property is expressed as


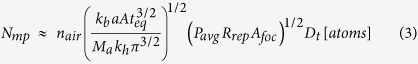


Thus, the pulse-to-pulse separation time is directly controlling the evaporation rate of the atoms, and similarly, the pulse repetition rate influences the number of evaporated atoms by a square root. To maintain the quality of these 3-D nanostructures and their overall repeatability, the pulse-to-pulse separation time is the controlling factor, where electron excitation and energy transfer occur in two separate phases. However, varying the repetition rate (MHz) of the ultrashort laser pulses results in bringing the temperature of the target surface to the temperature where the synthesized 3-D nanostructures are oxidized, depending on the ionization energy induced by this ultrashort laser pulses.

### Mechanism of multi-Ti oxide phased nanostructure formation by ultrashort pulsed laser

**Figure 2:**
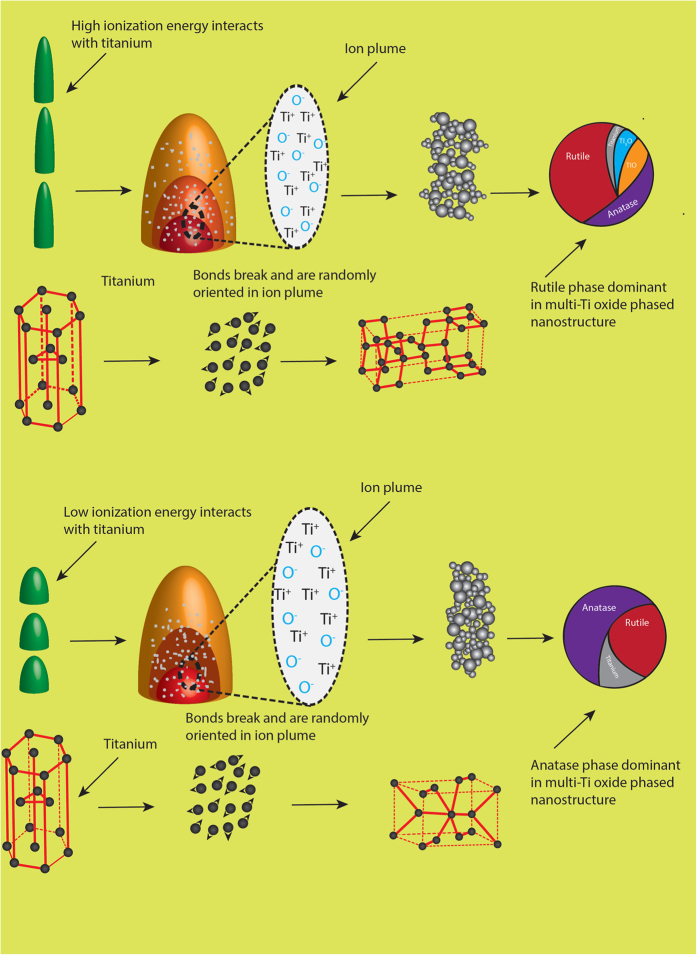
Graphical illustration of two dominant phases formed in a multi-Ti oxide phased nanostructure by varying the ionization energy of the ultrashort pulsed laser.

The mechanism of phase transformation in a material is fundamental for controlling the material characteristics of the synthesized nanostructure. Among the oxides formed, TiO_2_ is known for its polymorphism, and they exist in anatase, rutile and brookite phases. The stability of this polymorph is critical, and the kinetics of the parameters are essential to obtain multi-Ti oxide phased nanostructures. The phase transformation of anatase to rutile is invariable, and they begin to transform in the temperature range varying from 400–1200 °C^29^,^30^,^31^,^32^. The phase transformation of anatase to rutile is referred to as nucleation, and the growth process is dependent on variables such as heat flow condition, temperature and time. The thermodynamic phase stability of TiO_2_ polymorph is stable in the ascending order, anatase, brookite and rutile, which indicates that anatase and brookite are in less stable form when comparing with rutile^33^. This study shows that when the ionization energy is of higher magnitude, the titanium nanostructure formed is completely rutile phase dominant with the presence of the hongquiite phase. However, this hongquiite phase is not present when the ionization energy is of lower magnitude, resulting in the formation of anatase-dominant titanium nanostructures. This result indicates that at high energies and at very short condensation times, the in-between laser pulses results a higher amount of ionization energy, which results in a phase transformation of anatase into rutile (Fig. 2). Low energies and longer condensation times in-between pulses results in low ionization energies, leading to completely anatase phase dominant nanostructures^25^ Thus there remains a question as to why anatase in a less stable state is predominantly formed in TiO_2_, when the ionization energy is lower. Extensive research is needed to develop a clear understanding of how ionization energy plays an important role in determining phase transformation in nanostructures.

### Structural analysis and energy dispersive of multi-Ti oxide phased nanostructure

**Figure 3:**
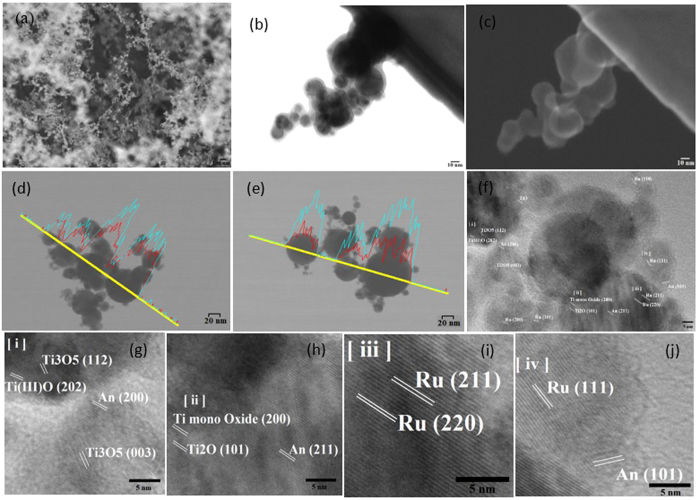
(**a**) FESEM (field emission scanning electron microscope) micrograph of multi Ti-oxide phase titanium nanostructure (**b**,**c**) Apparent core shell morphology of rutile-dominant nanostructure (**d**,**e**) TEM-EDX results of (**d**) anatase-dominant and (**e**) rutile-dominant nanostructure (**f**–**j**) HRTEM images of rutile-dominant nanostructures showing the different lattice spacing.

When the ionization energy from the ultrashort pulsed laser interacts with titanium material, this reveals the formation of 3-D multi-Ti oxide phased nanostructure; in this study, the arrangement and structural composition of multi-Ti oxide phased nanostructure is analyzed. A close-up view of a rutile-dominant nanostructure arrangement (Fig. 3(a)) indicates that it is an agglomeration of self-assembled randomly organized closed rings or chains that are connected by a small necking, as observed in Fig. 3(b,c). Individual nanoparticles are aggregated together and the mere loose packing in-between the nanoparticles is strong because of their bonds^25^. This structural arrangement of the nanostructure remains randomly arranged and displays no particular pattern. The back-scattered analysis of this rutile-dominant nanostructure reveals that the individual nanostructure is made up of two layers, an apparent core-shell morphology. Also this similar arrangement is also formed on anatase-dominant nanostructures. The TEM-EDX analysis reveals that there is variation in titanium and oxygen peaks in the individual nanostructures when the ionization energy is varied, and thus it is presumed that the outer shell is composed of various titanium oxides, which is validated by the XRD analysis. Anatase-dominant nanostructures are predominantly formed when the ionization energy is low (Fig. 3(d)), and the results obtained by TEM-EDX analysis provide evidence of the variation in titanium and oxygen peaks, which indicate there is formation of various phases of titanium oxides. However, when compared to anatase-dominant nanostructures, there is a significant variation in titanium and oxygen peaks of rutile-dominant nanostructures (Fig. 3(e)) that is formed due to higher ionization energy. For further verification, the distance between the lattice fringes was measured and identified for rutile-dominant nanostructures, which indicates there are diverse planes of anatase, rutile, titanium oxide (Ti_3_O_5_), and titanium mono oxide formed at when high ionization energy interacts with titanium material (Fig. 3(f–j)). This analysis confirms the mixed-oxide phases of titanium formed, which are further analyzed by the XRD analysis.

### Elemental characterization of multi-Ti oxide phased nanostructure by X-ray diffraction

**Figure 4:**
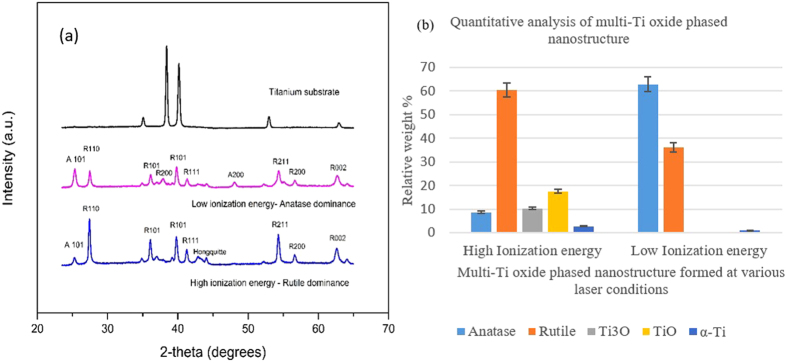
(**a**) XRD analysis of multi-Ti oxide phase nanostructures. (**b**) Quantitative analysis of various phases present in the nanostructures.

The elemental composition of this readily scalable multi-Ti oxide phased nano structure is quantitatively analyzed by employing XRD. This unique multi-Ti oxide phased nanostructure consists of tetragonal TiO_2_ (anatase and rutile) and cubic TiO (Hongquiite) unprocessed titanium substrate is entirely composed of alpha-phase titanium. In the previous section, a mixed proportion of these crystalline phases was observed by varying the ionization energy of the ultrashort pulsed laser. The random orientation of these crystal structures could be attributed to the varying plume mechanisms and available oxygen^32^. From Fig. 3(b), it observed that this multi-Ti oxide phase nanostructure has an apparent titanium core and shell morphology; this shell is made up of multiple titanium oxides, revealing the presence of anatase, rutile, trigonal oxide and non-stoichiometric cubic TiO phases. As previously discussed, the TEM-EDX analysis indicates there is a variation of titanium and oxygen peaks proportional the vapor condensation formation mechanism for anatase dominance nanostructure and rutile dominance nanostructure. The ratio of titanium and oxygen formation on this multi-Ti oxide nanostructure relates directly to high ionization energy, which is achieved at a high pulse fluence and the shortest pulse-to-pulse separation time, and similarly low ionization energy is achieved with a low fluence and the longest pulse-to-pulse separation time. As noted above as the peak power reduces with either an increase or decrease in the pulse-to-pulse separation time, the anatase phase is dominant over the rutile phase. However, only with the combination of high peak power and the shortest pulse-to-pulse separation time is the rutile phase dominant over the anatase phase and is accompanied by the presence of non-stoichiometric cubic TiO phase (Fig. 4). Also at high peak power and the longest pulse-to-pulse separation time, the rutile phase is dominant over the anatase phase, but there is no presence of rare titanium oxide phases (trigonal cubic TiO and non-stoichiometric cubic TiO). Thus, varying the pulse-to-pulse separation time at high peak power condition results in formation of rare titanium oxide phases. The role of these multi-Ti oxide phased nanostructures was further probed in the following section to study the potential correlation in reducing cancer cell differentiation and proliferation.

### Decreased HeLa and NIH3T3 cell adhesion on multi-Ti oxide phased nano structure

**Figure 5:**
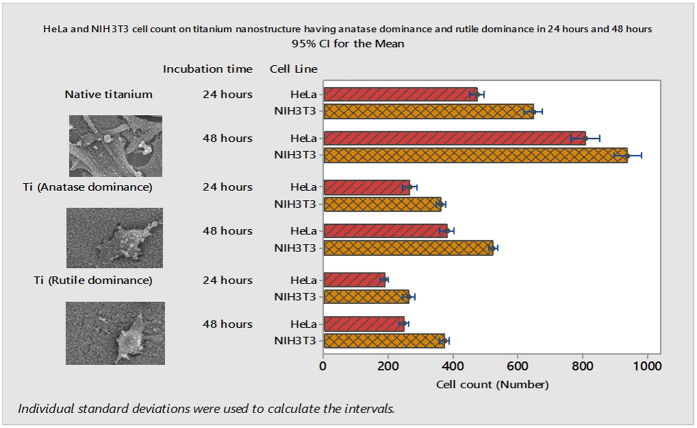
HeLa cell and NIH3T3 cell adhesion on anatase-dominant and rutile-dominant nanostructures at 24 hours and 48 hours.

**Figure 6:**
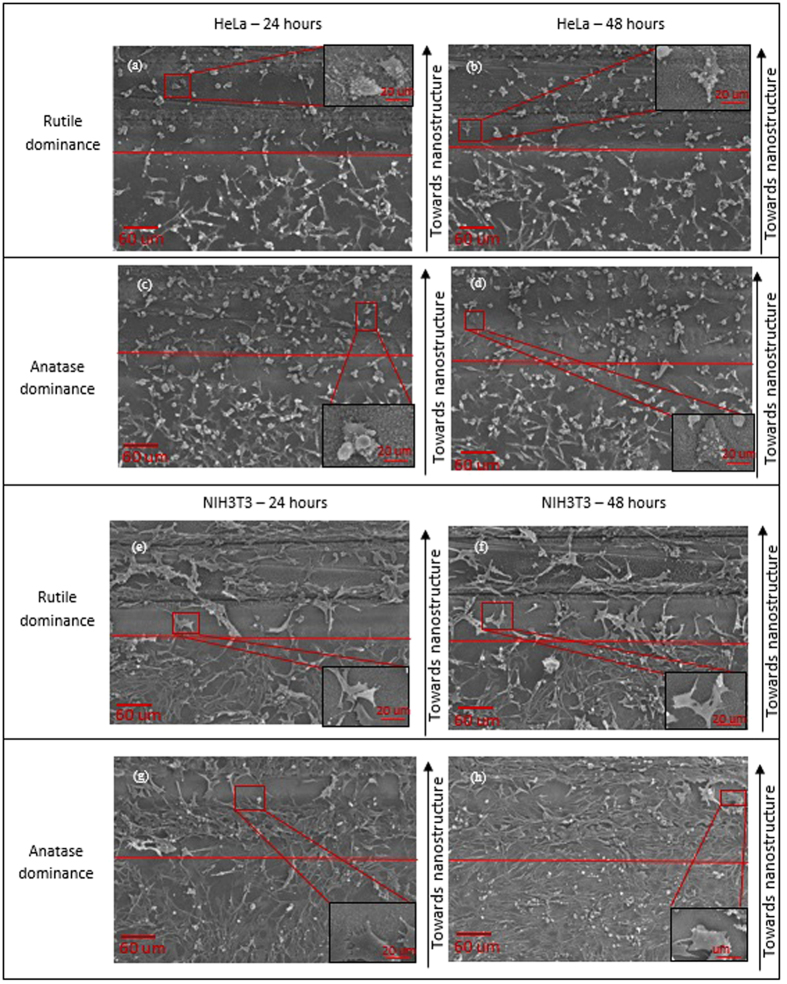
HeLa and NIH3T3 cells on anatase-dominant and rutile-dominant nanostructures at 24 hours and 48 hours.

The number of HeLa cells and the NIH3T3 cell adhesion on anatase-dominant and rutile-dominant nanostructures was investigated after incubation for 24 hours and 48 hours (Fig. 5). The cells cultured on smooth native titanium material were used as the control. To investigate if the anatase dominance or rutile dominance of multi-Ti oxide phase nanostructure reduces the HeLa cancer cell adhesion or supports survival of the NIH3T3 cells, we have quantified the total number of cells adhered onto the multi-Ti oxide phased nano structure at the end of 24 hours and 48 hours. The total number of the HeLa cells and the NIH3T3 cells adhered on rutile-dominant multi-Ti oxide phased nanostructure is greatly decreased when compared to anatase-dominant multi-Ti oxide phased nanostructure at the end of 24 hours (Fig. 6(a,c,e,g)). However, this reduction in cell adhesion was observed for both the HeLa cells and the NIH3T3 cells when they interact with the rutile-dominant multi-Ti oxide phased nanostructure, which indicates that these cell lines are susceptible to this dominant multi-Ti oxide phased nanostructure. These results substantiate the theory that cells sense changes in their environment and react via transmitting extracellular signals to the nucleus^34^. However, this order of magnitude decrease in the HeLa cell adhesion when compared to the fibroblast cells indicates that these fibroblast cells are less susceptible to anatase-dominant multi-Ti oxide phase nanostructures when compared to rutile-dominant nanostructures. At the end of 48 hours, the HeLa cells and the NIH3T3 cells we observed demonstrated an upsurge in cell proliferative characteristics when they interact with anatase-dominant nanostructures when compared to rutile-dominant titanium nanostructures (Fig. 6(b,d,f,h)). This outcome was the consequence of a multi-Ti oxide phased nanostructure losing their efficacy in controlling the cell proliferation. However, both the HeLa and the NIH3T3 cell proliferation in 48 hours was amassed in anatase-dominant nanostructure, when compared to rutile-dominant nanostructure, which divulge that these rutile-dominant nano structure are intrusive in controlling the cell proliferation. It could be speculated that at the rutile-dominant multi-Ti oxide phase nanostructure, the polymerization of actin filaments in the HeLa cells was adversely affected when compared to the anatase-dominant multi-Ti oxide phase nanostructure. Earlier Jung lu *et al.* reported that there is improved cellular adhesion when they interact with the nanostructure surfaces, which is achieved through by varying the height of the nanostructure^35^. Additionally, Popat *et al.* reported that there is evidence of enhanced cellular adhesion by synthesizing novel-metal oxide titania nanotubes that corroborate our research as the exclusive method in utilizing the dominant phases present in multi-Ti oxide phase nanostructure for modulating the cell adhesion^36^.

### Modulating HeLa cell cytoskeleton behavior when interacting on multi-Ti oxide phased nanostructure

**Figure 7:**
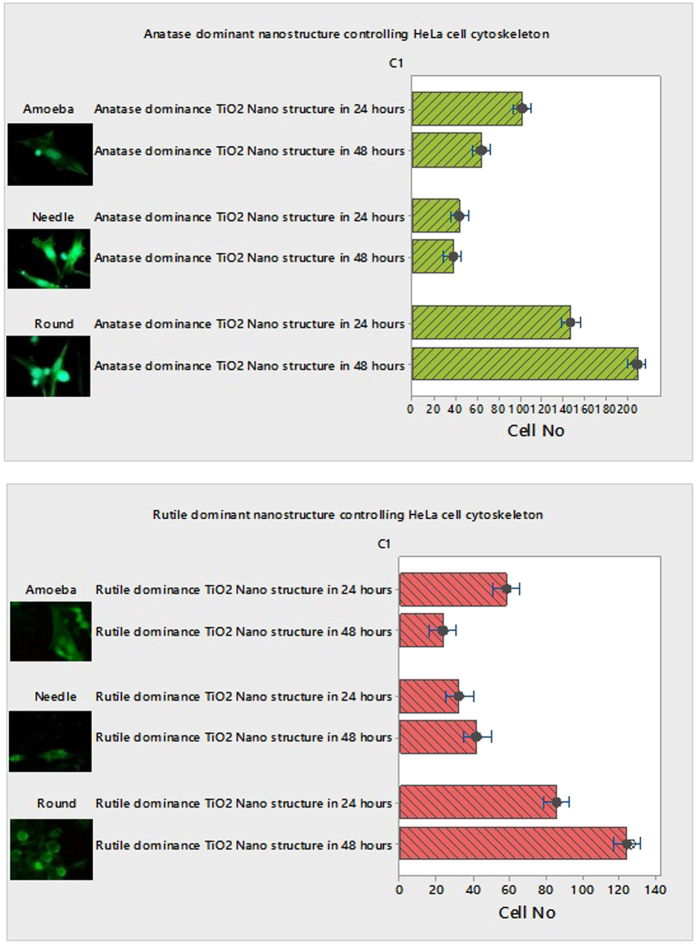
(1) Anatase-dominant nanostructure controlling HeLa cell cytoskeleton at 24 hours and 48 hours. (2) Rutile-dominant nanostructure controlling HeLa cell cytoskeleton behavior at 24 hours and 48 hours.

Using multi-Ti oxide phase nanostructures fabricated as described above, we are now analyzing how the HeLa cell cytoskeleton behavior is determined under the influence of phase dominance in nanostructures. After 24 hours the HeLa cell is predominantly amoeba shaped cells (well defined) found in higher number on the anatase-dominant nanostructures when compared to the rutile-dominant nanostructures (Fig. 7). We consistently found that the proportion of needle-shaped cells appeared in higher number on the rutile-dominant nanostructures when compared to the anatase-dominant nanostructures, which indicate that HeLa cells senses the change in material chemistry of the nanostructure favorable for them to migrate away and adhere. This behavior reveals that the HeLa cells do not favor the rutile-dominant nanostructure to adhere and form a well-defined shape (amoeba), which is contradictory to the HeLa cell interaction with the anatase-dominant nanostructure where amoeba shaped cell is in amassed magnitude^37^,^38^. However, to the best of our knowledge, there is no previous research on how the HeLa cell cytoskeleton behavior can be determined by phase dominant nanostructures. Our results corroborate to our earlier result, where cell proliferation is observed more on anatase-dominant nanostructures, which was also observed profusely.

### Lack of stress fiber on multi-Ti oxide phased nanostructure

**Figure 8:**
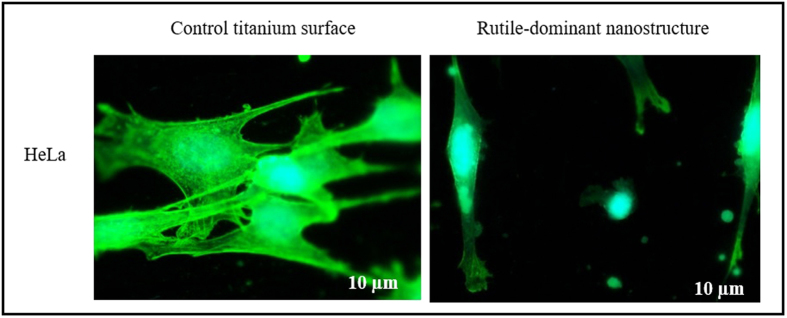
Fluorescent micrographs of HeLa cells on rutile-dominant nanostructures and control titanium surfaces.

When the HeLa cells adhered on multi-Ti oxide phased nanostructures, they were unable to form stress fibers, which implies that there is a decrease in average area of cell spread. This implies these nanostructures provides cues for cells not to initiate stress fibers because they are not the favorable location for the HeLa cells to adhere and proliferate (Fig. 8). However, no initiation of stress fibers in the HeLa cells, when they interact with both anatase-dominant and rutile-dominant nanostructures. This result indicates that both phase dominant nanostructures have influence in reducing the stress fiber formation in the HeLa cells. Also the HeLa cells have developed highly migratory morphology, having long protrusions, which is contrary to the control surface where cells appeared well spread with visible stress fibers that indicate they found a favorable place to adhere. Jiyeon Lee *et al.*, stated that when the spacing between the nanorods is more than 80–100 nm it results in non-adherence of cell onto the nanorods, which is contrary to our finding that the material chemistry of these multi-Ti oxide phased nanostructure results in determining actin stress fibers formation even when the distance between the nanostructure is less than 10 nm^39^. However, this similar reduction in stress fibers was also found when the cells adhered on zinc oxide nanorods where the diameter of the nanorods is approximately 50 nm^22^. When the HeLa cells adhered on multi-Ti oxide phased nanostructures they did not have visible lamellipodia, which is due to their inability to establish strong cell adhesion and thus it explains the decrease in weblike cell morphology and the increase round cell morphology on both phase dominant nanostructures^40^.

### AFM analysis of multi-Ti oxide phased nanostructure interacting with HeLa cells

**Figure 9:**
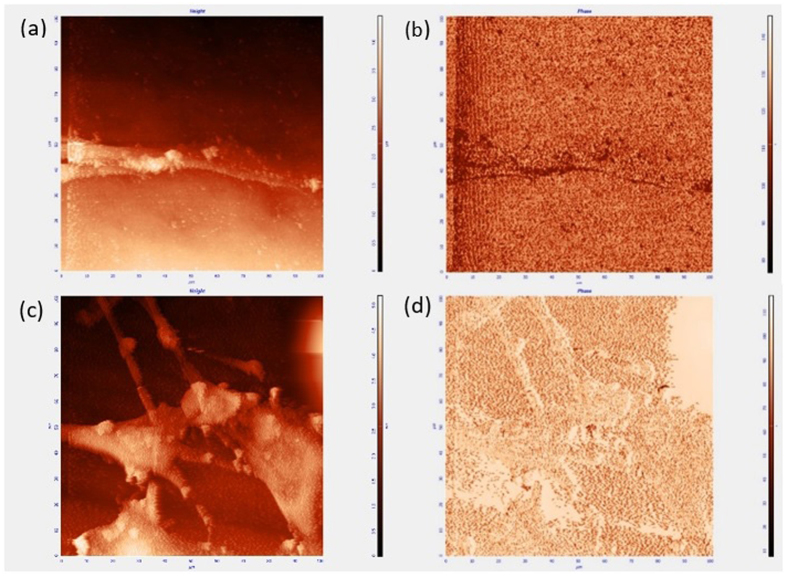
AFM method to analyze the height and phase contrast image of the HeLa cancer cells interacting with rutile-dominant nanostructures and the control sample. (**a**) Rutile-dominant nanostructure with the HeLa Cell – Height (**b**) Rutile-dominant nanostructure with the HeLa cell – Phase (**d**) Control sample with the HeLa cell – Height (**e**) Control sample with the HeLa cell - Phase.

AFM is the only method we can use to understand the extracellular structures of the cell organized at various surfaces, chemical compositions and physiochemical properties of the nanostructures they interact at the same time. The Fig. 9(a,b) provides evidence as to how the HeLa cells when interacting with rutile-dominant nanostructures give rise to a complex phase signal shift in the sample. These phase contrast images provides valuable information on the heterogeneity of the rutile-dominant nanostructures, which is the evidence to X-ray diffraction analysis that the rutile-dominant nanostructure synthesized is a mixture of anatase, rutile, trigonal oxide and non-stoichiometric cubic TiO phases. Figure 9(c) shows the cells adhered to the control titanium sample and cells form broad lamellipodia, which is evidence that the HeLa cells adhered firmly because they have found a favorable place to adhere and proliferate. In the phase contrast image in Fig. 9(d), there is no evidence of variations in material properties, but in Fig. 9(b) there are variations in material property. These results indicate that the HeLa cells elongate, when there is a variation in material property, but broad lamellipodia are formed, when there is no variation in material property. Thus, the AFM analysis of multi-Ti oxide phased nanostructure interacting with the HeLa cells is the method where the HeLa cell behavioral changes due to the change in material property can be analyzed simultaneously.

### Surface area analysis of HeLa cell on multi-Ti oxide phased nano structure

**Figure 10:**
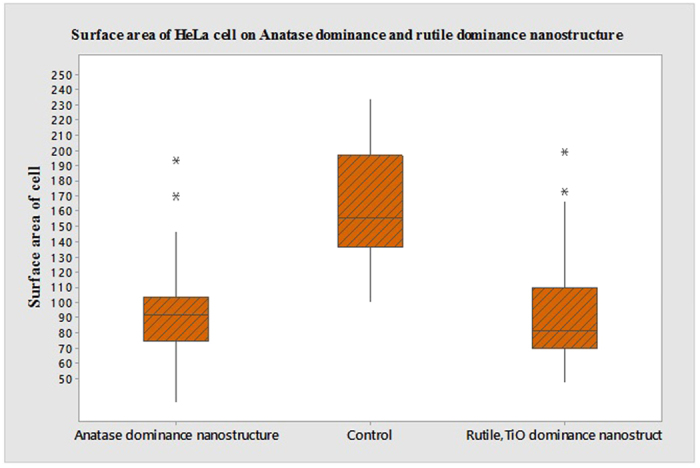
Surface area of HeLa cell on anatase-dominant nanostructure and rutile-dominant nanostructure and control titanium sample in 24 hours, units in μm^2^.

On the smooth control surface, the HeLa cells exhibited multipolar morphology with wide distribution in surface area measurement for the HeLa cells. In the anatase-dominant nanostructures, both amoeba and needle shaped cells were seen predominantly when compared to the rutile-dominant nanostructures. Thus, when the composition of the nanostructure is varied and applied to the HeLa cell cytoskeleton study, there is evidence in varied cytoskeleton morphology depending on the dominant nanostructure they interact (Fig. 7). The quantified results for the surface area measurement of the HeLa cells are presented in the box whisker plots (Fig. 10). The surface area measurement graph displayed the HeLa cell distribution midpoint (median), the first and third quartile (boxes), and the largest and smallest observation (whiskers). The results on the smooth control surface showed that the median for surface area of the cell is 160 μm^2^. When subjected to rutile-dominant nanostructures, the surface area of the cells gradually decreased, and the median is 80.4 μm^2^. However, in the anatase-dominant nanostructures, the surface area of cells median is 91 μm^2^, which indicates that the cells do not prefer to adhere on rutile-dominant nanostructures. Thus, the most probable occurrence of surface area is high on anatase-dominant nanostructures when compared to rutile-dominant nanostructures, even when they both have same morphology.

## Conclusion

Current research has shown that the morphology of the nanostructure or single oxide present in the nanostructure controls the proliferative characteristics of cells. However, in this study the HeLa cells and NIH3T3 cells were cultured on multi-Ti oxide phase nanostructures for studying their cell adhesion and cytoskeleton behavior. The results added a new insight into how fibroblast cells behave differently when compared to HeLa cells, depending on the dominant phase present in the nanostructure they interact with. However, this unique interaction behavior of cells with nanostructures having dominant phases provided an opportunity to understand how the composition of the individual oxide phases can be tuned depending on the specific application. The ultrashort femtosecond pulsed laser method proved to be a versatile technique by allowing us to program the composition of titanium oxide phases in a nanostructure that enables us to manipulate the HeLa cancer cells scrupulously but seldom (NIH3T3) fibroblast cells is the highlight of this research. There have been no previous reports on synthesizing multi-Ti oxide phases in a nanostructure and their intelligence to modulate the HeLa and NIH3T3 cells differently. Thus, the application of this multi-Ti oxide phased nanostructure has the potential applications of tissue engineering, implant coatings and point of care diagnostic devices.

## Additional Information

**How to cite this article**: Chinnakkannu Vijayakumar, C. *et al.* Harmonizing HeLa cell cytoskeleton behavior by multi-Ti oxide phased nanostructure synthesized through ultrashort pulsed laser. *Sci. Rep.* **5**, 15294; doi: 10.1038/srep15294 (2015).

## Figures and Tables

**Figure 1:**
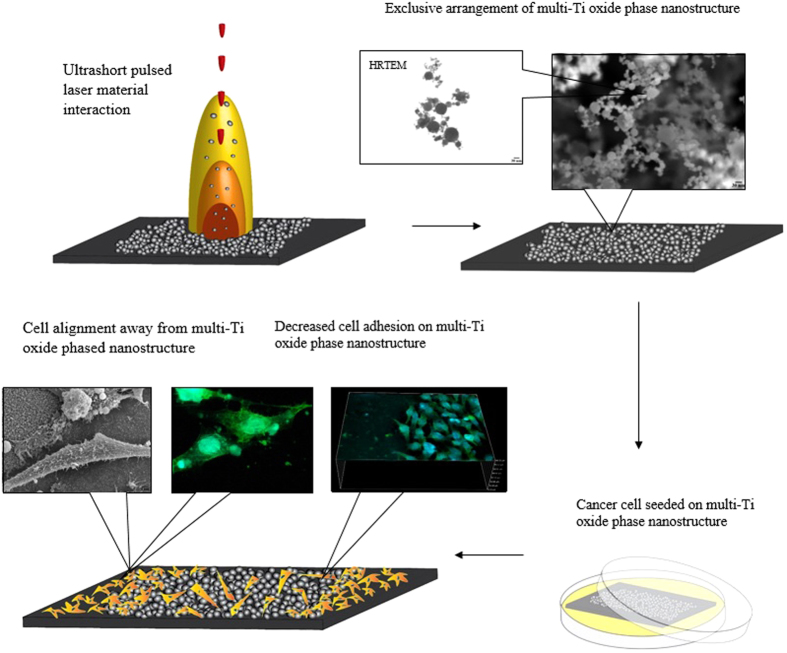
Overall graphical abstract for synthesizing multi-Ti oxide phased nanostructure and its ability to modulate HeLa cell cytoskeleton behavior.
